# Upregulation of deubiquitinase *UBP16 *induced by rice stripe virus infection stabilizes SHMT1 to suppress ROS accumulation and facilitate virus infection in *Nicotiana benthamiana*

**DOI:** 10.1007/s44154-025-00265-2

**Published:** 2025-10-26

**Authors:** Kun Wang, Yaqin Wang, Shuai Fu, Yuchong Tan, Liang Wu, Yi Xu, Jianxiang Wu, Xueping Zhou

**Affiliations:** 1 State Key Laboratory of Rice Biology and Breeding, College of Agriculture and Biotechnology, Zhejiang University, Hangzhou, 310058 China; 2 State Key Laboratory for Biology of Plant Diseases and Insect Pests, Institute of Plant Protection, Chinese Academy of Agricultural Sciences, Beijing, 100193 China; 3 Department of Plant Pathology, Nanjing Agricultural University, Nanjing, 210095 China

**Keywords:** Rice stripe virus, NbUBP16, Ubiquitination, SHMT1, ROS accumulation

## Abstract

Modification of proteins by ubiquitin is a dynamic and reversible process. It is unclear whether rice stripe virus (RSV) can modulate the plant deubiquitination pathway. In this study, we found that RSV infection can specifically upregulate the expression of the deubiquitinase *NbUBP16*. Further analysis revealed that NbUBP16 stabilizes serine hydroxymethyltrasferase (SHMT1) by binding to NbSHMT1 and removing its polyubiquitination modification mediated by E3 ligase MEL, which inhibits downstream SHMT1-mediated ROS accumulation and thereby facilitates RSV infection. Our findings provide new insights into the molecular arms race between pathogens and plants, demonstrating how a plant virus can undermine plant defenses by hijacking host deubiquitination pathways.

**Supplementary Information:**

The online version contains supplementary material available at 10.1007/s44154-025-00265-2.

## Introduction

As one of the most widely cultivated and consumed grains, rice (*Oryza sativa* L.) plays a critical role in food security and economic stability in many regions (Hickey et al. 2019). The production and quality of rice are therefore of paramount importance, with any threats to its yield, such as disease and pest pressures, posing significant risks to global food supplies (Savary et al. 2019).

Rice stripe virus (RSV) is one of the most destructive viruses in rice production and seriously threatens rice yield (Xu et al. 2021). RSV belongs to the *Tenuivirus* genus, and its genome consists of four single-stranded RNA segments (RNAs 1, 2, 3, and 4). RSV is transmitted between plants by the small brown planthopper (*Laodelphax striatellus*) in a circulative-propagative manner. It also can be transmitted transovarially by viruliferous female planthoppers to their offspring through eggs (Xu et al. 2021).

In the long process of evolution, plants and pathogens have developed intricate attack and defense strategies (Savary et al. 2019). Plants establish their immune barrier primarily through two layers of defense responses: PAMP-triggered immunity (PTI) and effector-triggered immunity (ETI), leading to a series of cellular and physiological responses, including the accumulation of reactive oxygen species (ROS) and up-regulation of disease-related gene expression (Yu et al. 2017; Yuan et al. 2021; Ngou et al. 2022). In turn, pathogens have evolved sophisticated effectors to suppress plant defense responses and facilitate infection (Huang et al. 2023; Wang et al. 2023a; Zhang et al. 2023).

Serine hydroxymethyltransferase (SHMT) is an essential enzyme found across all living organisms, catalyzing the reversible conversion of serine and tetrahydrofolate (THF) to glycine and 5,10-methylene THF, thus supplying one-carbon units necessary for thymidylate, purine, and methionine synthesis (Ducker and Rabinowitz 2017). SHMT plays a central role in one-carbon metabolism and reactive oxygen species (ROS) production, which together modulate cellular responses to diverse stresses, including biotic and abiotic stresses (Zhou et al. 2012; Gupta et al. 2017). In plants, SHMT exists as paralogs that localize to various cellular compartments, such as the cytosol, mitochondria, plastids, and nucleus, each potentially contributing to specialized metabolic functions within these regions (Zhang et al. 2010; Zhou et al. 2012). Previously, our lab identified an evolutionarily conserved E3 ligase, MEL, which can ubiquitinate and degrade SHMT1, causing the accumulation of hydrogen peroxide, thereby conferring plants with broad-spectrum resistance to various pathogens (Fu et al. 2022). Our further study revealed that RSV encoded NS3 protein can specifically bind to the substrate recognition domain of MEL, disrupting the plant MEL-SHMT1-mediated immune response in plants (Wang et al. 2023b).

The deubiquitinating enzymes (DUBs) hydrolyzes the covalent bond at the end of ubiquitin to specifically remove ubiquitin molecules from proteins linked with ubiquitin, thereby playing an important role in deubiquitination and regulating protein stability and function (Komander et al. 2009; March and Farrona 2018; Luo et al. 2023). Previously, many studies have demonstrated that plant deubiquitinases are involved in regulating plant development and stress responses (Zhou et al. 2012; Zhao et al. 2013; Li et al. 2020). However, whether plant deubiquitinase, such as UBP16, can respond to and regulate plant virus infection remains unknown.

Here, we found that RSV infection can specifically activate the upregulation of *NbUBP16* expression. NbUBP16.1 can interact with NbSHMT1 and remove polyubiquitination modification of NbSHMT1 to stabilize NbSHMT1 and inhibit ROS burst, thereby promoting RSV infection. This study further reveals the mechanism by which viruses promote viral infection by manipulating host factors.

## Results

### *NbUBP16* responds to and regulates RSV infection

**Fig. 1:**
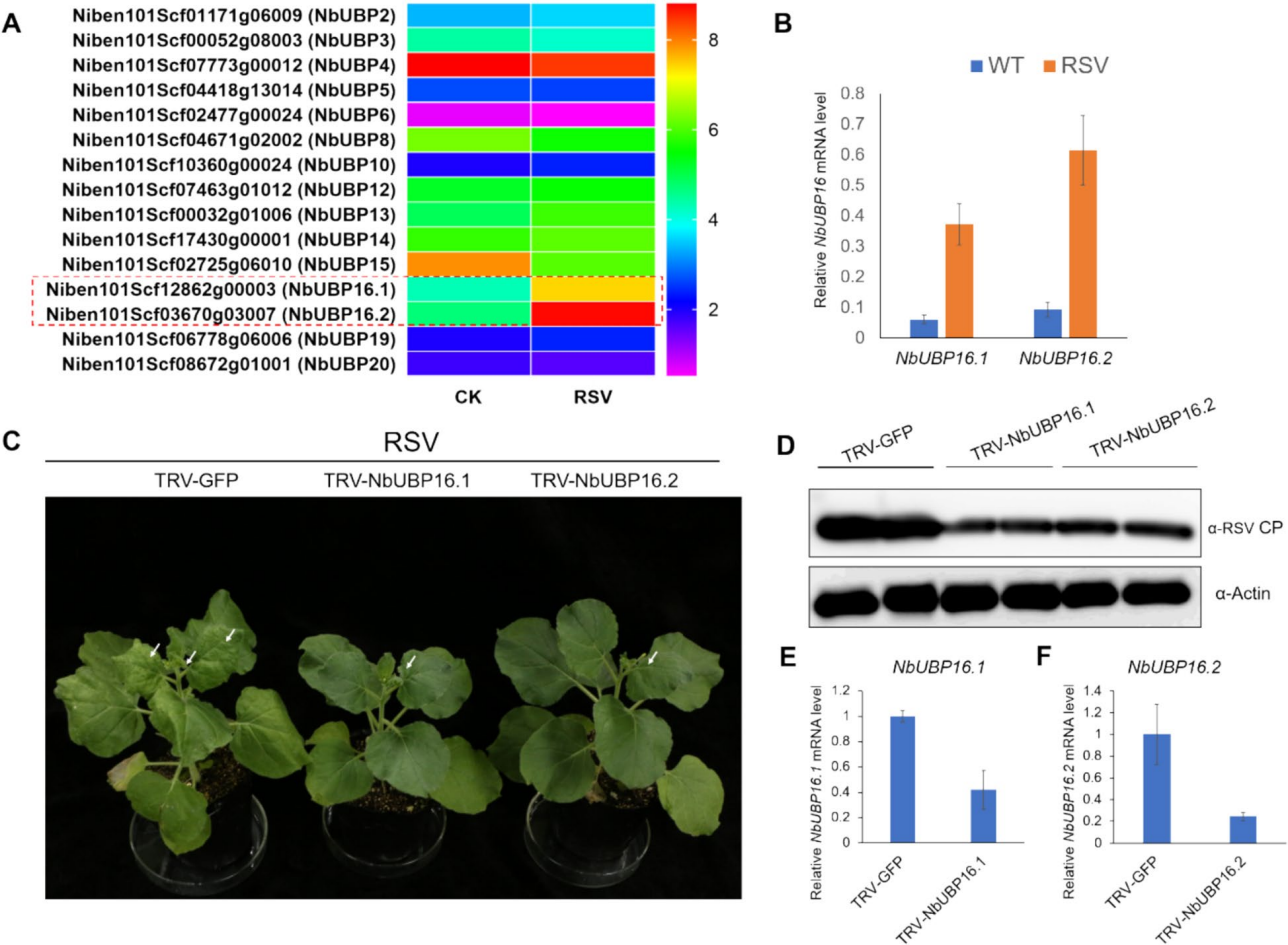
*NbUBP16* responds to and regulates RSV infection. **A** Heat-map for 15 NbUBP transcripts identified by RNA-seq. Relative expression values ranged from 0 to 10 were indicated by color bars. **B** Deubiquitinase *NbUBP16.1* (*Niben101Scf12862g00003.1*) and *NbUBP16.2* (*Niben101Scf03670g03007.1*) transcript levels in mock (WT) or RSV infected *N. benthamiana* systemic leaves at 10 dpi detected by qRT-PCR. Data are mean ± SD (*n* = 3). **C** Silencing of *NbUBP16* suppresses RSV infection. Photographs of representative symptoms were taken at 13 dpi. **D** RSV accumulation in *NbUBP16-*silenced and control (TRV-GFP) *N. benthamiana* plants infected by RSV at 13 dpi. Actin was used as loading controls. **E** and **F** Silencing efficiency of *NbUBP16.1* (**E**) and *NbUBP16.2* (**F**) detected by qRT-PCR. Data are mean ± SD (*n* = 3). Actin was used as a reference gene

To investigate whether RSV infection can impact the plant deubiquitination pathway, we analyzed RNA-sequencing data of RSV-infected and mock-inoculated (CK-) *Nicotiana benthamiana* plants at 10 days post-inoculation (dpi) to identify differentially expressed deubiquitinases. The results revealed that 11 transcripts showed no significant difference among the 15 analyzed transcripts. However, two transcripts, *Niben101Scf02725g06010 (NbUBP15)* and *Niben101Scf08672g01001 (NbUBP20)* were down regulated, while *Niben101Scf12862g00003 (NbUBP16.1)* and its homologs *Niben101Scf03670g03007 (NbUBP16.2)* were notably up-regulated (Fig. 1A). *NbUBP16.1* and *NbUBP16.2* have 96% sequence similarity and are highly homologous at the 5' and 3' ends of the coding regions. In addition, reverse transcription-quantitative PCR (RT-qPCR) analysis of *N. benthamiana* plants inoculated with RSV via mechanical inoculation using infected rice leaves as inoculum showed that the expression of *NbUBP16.1* and *NbUBP16.2* increased by approximately 4 to 6-fold at 10 dpi in RSV-systemically infected leaves compared to plants inoculated with healthy rice leaves (Fig. 1B). Furthermore, silencing of *NbUBP16.1* or *NbUBP16.2* via tobacco rattle virus (TRV)-induced gene silencing, followed by RSV inoculation, demonstrated that knock-down of these transcripts significantly suppressed RSV symptom development and reduced viral capsid protein (CP) accumulation (Fig. 1C and D). The silencing efficiencies of *NbUBP16.1* and *NbUBP16.2* are about 60% and 70%, respectively (Fig. 1E and F). These findings indicate that *NbUBP16* responds to RSV infection and may play a role in regulating viral infection.

### NbUBP16.1 interacts with NbSHMT1 but not with E3 ligase NbMEL

**Fig. 2:**
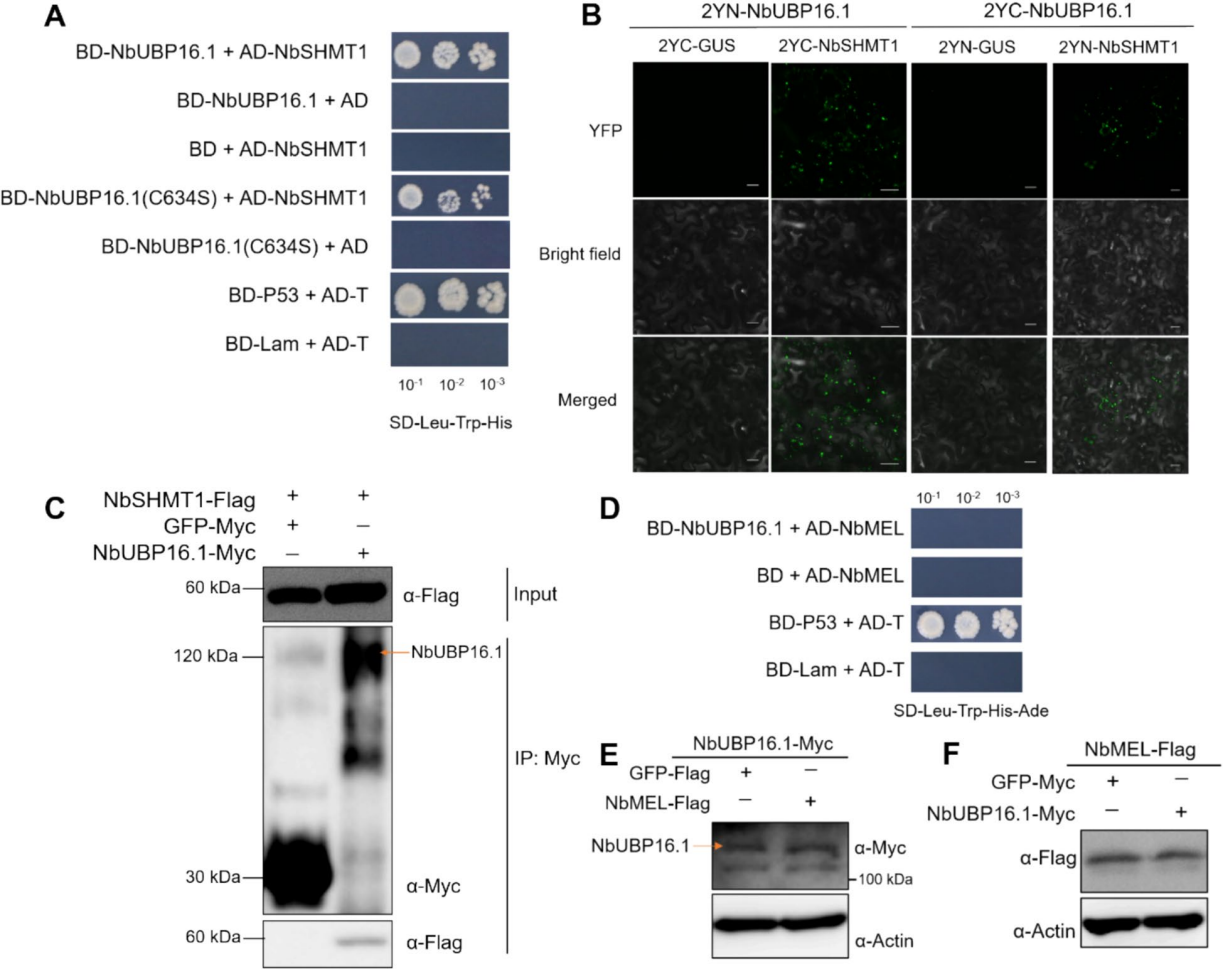
Deubiquitinase NbUBP16.1 interacts with NbSHMT1 but not with E3 ligase NbMEL. **A** NbUBP16.1 interacts with NbSHMT1 in yeast two hybrid assay. **B** NbUBP16.1 interacts with NbSHMT1 by BiFC assay. Confocal images were taken in *N. benthamiana* epidermis tissue at 48 hpi. Bars: 20 μm. **C** Co-immunoprecipitation analysis of the interaction between NbUBP16.1 and NbSHMT1. Myc beads were used to enrich NbUBP16.1-Myc and GFP-Myc. **D** NbUBP16.1 does not interact with NbMEL in yeast two hybrid assay. **E** In vivo protein stability assay of the effect of NbMEL on the stability of NbUBP16.1 in *N. benthamiana* plants. **F** In vivo protein stability assay of the effect of NbUBP16.1 on the stability of NbMEL in *N. benthamiana* plants. GFP was used a negative control. Actin was used as loading controls

To explore how *NbUBP16* regulates RSV infection, we attempted to identify its interacting proteins. However, due to the high homology of the 5' and 3' ends of the coding regions of *NbUBP16.1* and *NbUBP16.2*, we only amplified the full-length *NbUBP16.1* fragment from *N. benthamiana*. Previous studies by Zhou et al. have demonstrated that *AtUBP16* plays a crucial role in plant salt stress response, specifically through the deubiquitination and stabilization of AtSHMT1 (Zhou et al. 2012). The sequence homology analysis results show that *NbUBP16.1* and *AtUBP16* have 72% sequence homology. Given the high homology between NbUBP16.1 and AtUBP16, we hypothesized that NbUBP16.1 may also interact with NbSHMT1. To test this, we constructed yeast two-hybrid (Y2H) vectors for both NbUBP16.1 and NbSHMT1 to verify their interaction. The Y2H assay revealed that NbUBP16.1 could interact with NbSHMT1 (Fig. 2A). Furthermore, co-immunoprecipitation (Co-IP) and bimolecular fluorescence complementation (BiFC) assays further confirmed that interaction between NbUBP16.1 and NbSHMT1 in plants (Fig. 2B and C). The BiFC analysis specifically indicated that NBUBP16.1 interacts with NbSHMT1 in the cytoplasm, forming distinct dot-like structures (Fig. 2B). Additionally, we previously reported that the E3 ligase NbMEL can interact with and ubiquitinate SHMT1 (Fu et al. 2022). We further tested the possibility of an interaction between the E3 ligase NbMEL and the NbUBP16.1, and our result showed that NbMEL did not interact with NbUBP16.1 (Fig. 2D). Moreover, NbMEL and NbUBP16.1 do not affect each other's protein stability (Fig. 2E and F).

### NbUBP16.1 removes the ubiquitination modification of NbSHMT1 mediated by E3 ligase NbMEL and stabilizes NbSHMT1

**Fig. 3:**
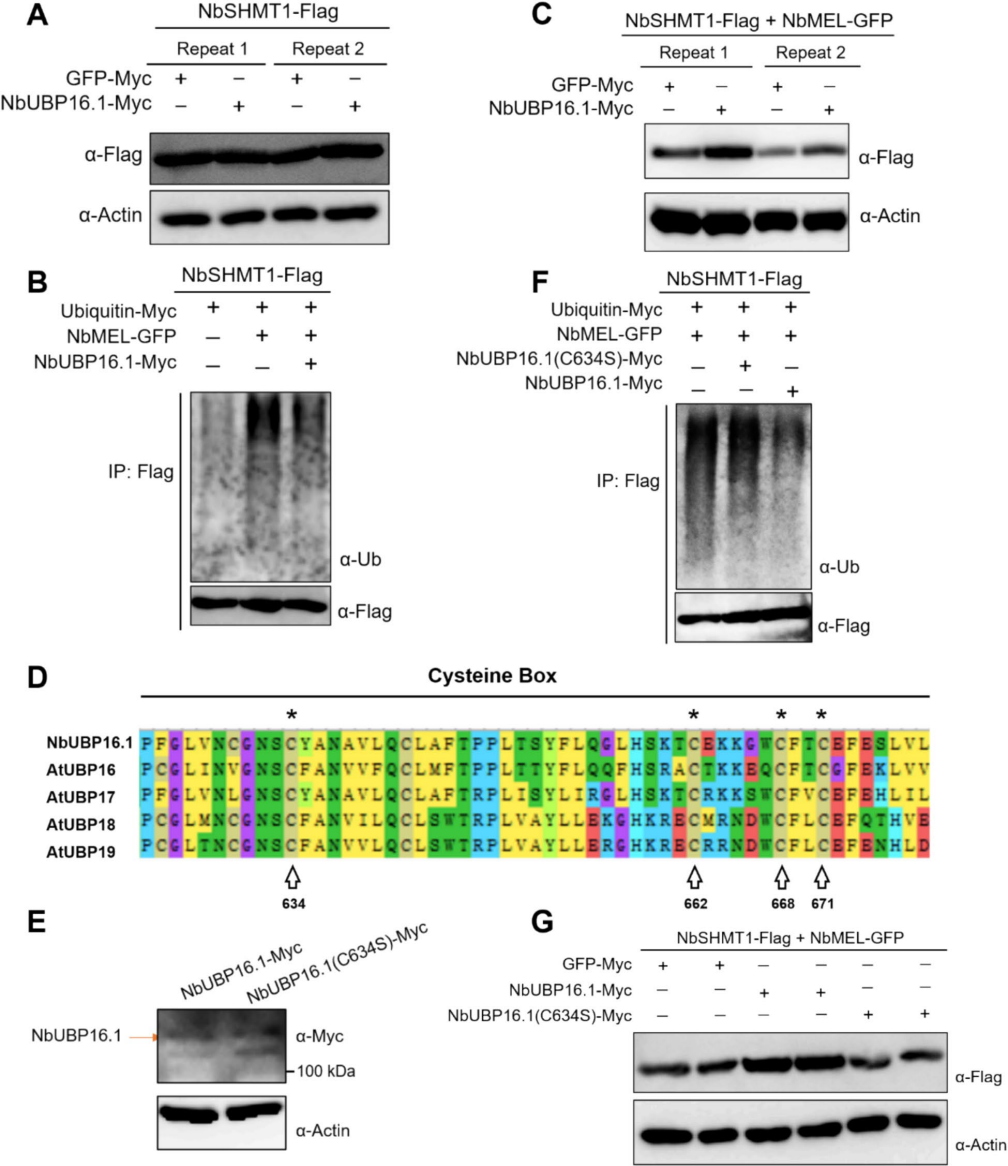
NbUBP16.1 removes the ubiquitination modification of NbSHMT1 mediated by E3 ligase NbMEL and stabilizes NbSHMT1. **A** In vivo protein stability assay of the effect of NbUBP16.1 on the stability of NbSHMT1 in *N. benthamiana* plants. **B** In vivo deubiquitination of NbSHMT1 by NbUBP16.1. NbMEL and NbUBP16.1 were added to the corresponding combinations, respectively. NbSHMT1 alone was used as a negative control. **C** Effect of NbUBP16.1 on the stability of NbSHMT1 in the background of expressing E3 ligase NbMEL in *N. benthamiana* plants. GFP was used a negative control. **D** Analysis of amino acid conservation of cysteine box of NbUBP16.1, AtUBP16, AtUBP17, AtUBP18 and AtUBP19. **E** Comparison of expression level of NbUBP16.1 and NbUBP16.1(C634S). **F** In vivo deubiquitination of NbSHMT1 by NbUBP16.1 and NbUBP16.1(C634S). NbUBP16.1 and NbUBP16.1(C634S) were added to the corresponding combinations, respectively. Co-expression of NbMEL and NbSHMT1 were used as positive controls. **G** Effect of NbUBP16.1 or NbUBP16.1(C634S) on the stability of NbSHMT1 in *N. benthamiana* plants. GFP was used a negative control. Actin was used as loading controls

Given that NbUBP16.1 contains a ubiquitin hydrolase domain, we try to explore whether it can promote the deubiquitination modification of NbSHMT1. Firstly, we conducted In vivo protein stability assays to detect whether NbUBP16.1 affects the stability of NbSHMT1. The results showed that NbUBP16.1 alone had no significant effect on NbSHMT1 protein accumulation (Fig. 3A). Next, we performed an In vivo ubiquitination assay. The results revealed that the ubiquitination level of NbSHMT1, when expressed transiently in plants, was relatively low. However, co-expression of E3 ligase NbMEL significantly increased the ubiquitination of NbSHMT1. However, the addition of NbUBP16.1 significantly reduced the polyubiquitination level of NbSHMT1, indicating that NbUBP16.1 can promote the deubiquitination modification of NbSHMT1 (Fig. 3B). Moreover, the results of protein stability assay revealed that, when co-expressing NbMEL and NbSHMT1 in plants, compared with the control GFP, the expression of NbUBP16.1 significantly enhanced the accumulation of NbSHMT1 protein (Fig. 3C). Cysteine-box of the ubiquitin-specific protease (USP) enzymatic core plays an important role in the process of removing ubiquitin from ubiquitinated substrates. We then performed sequence homology analysis with AtUBP16/17/18/19 and generated an enzyme activity-deficient mutant, NbUBP16.1(C634S), for functional validation (Fig. 3D). There was no significant difference in protein accumulation between NbUBP16.1 and NbUBP16.1(C634S) mutant (Fig. 3E). The Y2H assay demonstrated that NbUBP16.1(C634S) retained the ability to interact with NbSHMT1 (Fig. 2A). However, the results of In vivo ubiquitination assay showed that compared with NbUBP16.1, which effectively promotes the deubiquitination of NbSHMT1, the NbUBP16.1(C634S) mutant exhibited a significantly reduced ability to deubiquitinate NbSHMT1 (Fig. 3F). Additionally, In vivo protein stability assay showed that, in contrast to NbUBP16.1, the ability of NbUBP16.1(C634S) to stabilize NbSHMT1 was significantly reduced (Fig. 3G). These findings indicate that NbUBP16.1 can remove the poly-ubiquitination of NbSHMT1 mediated by E3 ligase NbMEL and stabilize NbSHMT1.

### OE-NbSHMT1 represses plant defense responses and promotes RSV infection

**Fig. 4:**
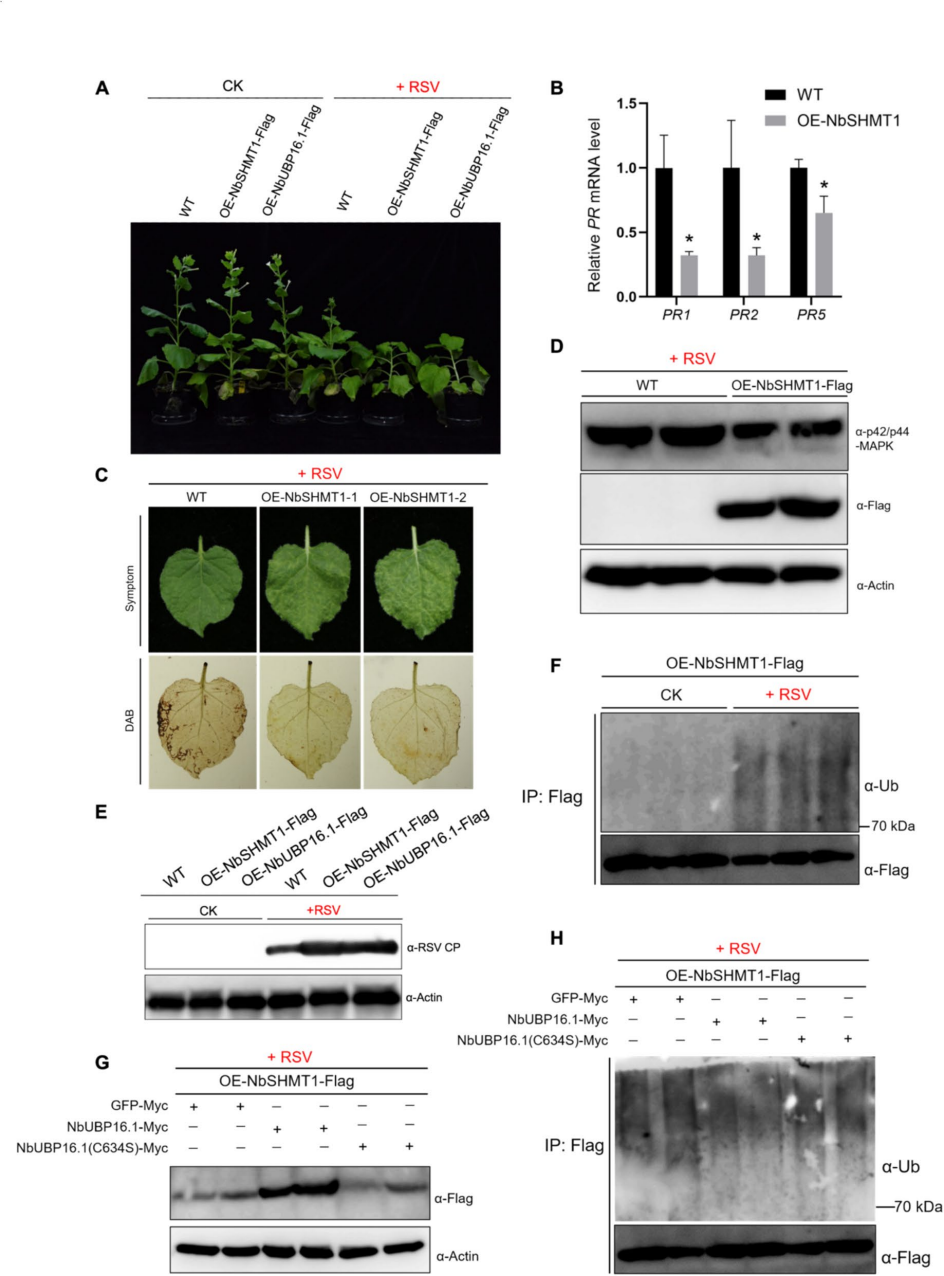
OE-NbSHMT1 represses plant defense responses and promotes RSV infection. **A** Photographs of representative RSV symptoms in *NbSHMT1* (OE-NbSHMT1) or *NbUBP16.1* overexpressing transgenic (OE-NbUBP16.1) and wild type *N. benthamiana* plants after RSV infection at 27 dpi. **B** Detection of transcripts of pathogen-related gene, including *PR1*, *PR2* and *PR5*, in wild-type and OE-NbSHMT1 *N. benthamiana* plants after RSV infection at 5 dpi. Actin was used as a reference gene. **C** DAB staining of wild-type and OE-NbSHMT1 *N. benthamiana* leaves after RSV infection at 15 dpi. **D** MAP kinase phosphorylation detection in wild-type and OE-NbSHMT1 *N. benthamiana* plants after RSV infection at 5 dpi. **E** RSV CP accumulation in wild-type, OE-NbSHMT1 and OE-NbUBP16.1 *N. benthamiana* plants infected by RSV at 27 dpi. Actin was used as loading controls. **F** Detection of ubiquitination level of NbSHMT1 in OE-NbSHMT1 transgenic *N. benthamiana* plants after RSV infection at 7 dpi. Flag beads were used to enrich NbSHMT1-Flag. Ub antibody was used to detect the ubiquitination level of NbSHMT1. **G** In vivo deubiquitination of NbSHMT1 by NbUBP16.1 and NbUBP16.1(C634S) in OE-NbSHMT1 transgenic *N. benthamiana* plants after RSV infection at 15 dpi. Flag beads were used to enrich NbSHMT1-Flag. Ub antibody was used to detect the ubiquitination level of NbSHMT1. **H** Effect of NbUBP16.1 or NbUBP16.1(C634S) on the stability of NbSHMT1 in OE-NbSHMT1 transgenic *N. benthamiana* plants after RSV infection at 15 dpi. GFP was used a negative control. Actin was used as loading controls

Next, we aimed to investigate whether NbSHMT1 could regulate plant defense responses against RSV infection, we constructed overexpression vectors for NbSHMT1, incorporating them into the pCambia vector to generate 2 × 35S-NbSHMT1-Flag vectors. This vector was then transformed into *N. benthamiana* plants. The successful integration of the transgenes was confirmed by RT-PCR (Fig. S1), and the expression of NbSHMT1 protein was verified by immunoblotting (Fig. S2). The growth of OE-NbSHMT1 transgenic plants were morphologically indistinguishable from wild-type *N. benthamiana* plants under normal conditions (Fig. 4A). This is consistent with the phenotype of OE-OsSHMT1 transgenic rice (Fu et al. 2022).

Given that OsSHMT1 negatively regulates rice defense responses, we aimed to investigate whether NbSHMT1 represses plant defense responses. The results revealed that the transcript levels of *PR1*, *PR2* and *PR5* genes were significantly reduced in OE-NbSHMT1 transgenic plant inoculated with RSV (Fig. 4B). The results of DAB staining revealed that, compare with wild-type *N. benthamiana* plants, OE-NbSHMT1 does not show significant difference in ROS accumulation under normal conditions (Fig. S3). However, OE-NbSHMT1 transgenic plant exhibited remarkably reduced ROS accumulation following RSV inoculation compared with wild-type *N. benthamiana* plants (Fig. 4C). Moreover, the result of the MAPK pathway activation assay showed that the MAPK pathway was significantly inhibited in OE-NbSHMT1 transgenic plant inoculated with RSV (Fig. 4D). These results demonstrated that NbSHMT1 negatively regulates plant defense responses.

To determine whether NbSHMT1 can regulate RSV infection, OE-NbSHMT1 and wild-type *N. benthamiana* plants were challenged with RSV. The results showed that, compared with wild-type plants, OE-NbSHMT1 transgenic plants exhibited enhanced viral symptoms and increased accumulation of the RSV capsid protein (Fig. 4A and E). To detect the ubiquitination level of NbSHMT1 after RSV infection, proteins were extracted from OE-NbSHMT1 plants at 7 days after inoculation with healthy or RSV-infected rice leaves as inoculum. Flag beads were used to enrich NbSHMT1-Flag and Ub antibody was used to detect the ubiquitination level of NbSHMT1. The results revealed that the ubiquitination level of NbSHMT1 increased significantly after RSV infection as compared with noninfected control treatment (Fig. 4F).

Next, to investigate whether NbUBP16.1 can affect the polyubiquitination of NbSHMT1 caused by RSV infection, *Agrobacterium tumefaciens* EHA105 strains containing NbUBP16.1, NbUBP16.1(C634S) mutant or control GFP vector were transiently expressed in the OE-NbSHMT1 transgenic plants 15 days after RSV inoculation, and the ubiquitination level and protein accumulation of NbSHMT1 were detected 48 h after agrobacterium inoculation. The results showed that, compared with the control GFP and NbUBP16.1(C634S) mutant, NbUBP16.1 could inhibit the polyubiquitination level of NbSHMT1 and promote the accumulation of NbSHMT1 protein (Fig. 4G and H). Moreover, we detected the difference of *NbMEL* and *NbUBP16.1* expression at different time points after RSV infection. The results showed that *NbMEL* expression is upregulated prior to *NbUBP16.1* after RSV infection (Fig. S4). These results demonstrated that NbUBP16.1 could inhibit the polyubiquition level of NbSHMT1 caused by RSV infection and stabilize NbSHMT1.

### NbUBP16.1 positively regulates RSV infection

**Fig. 5:**
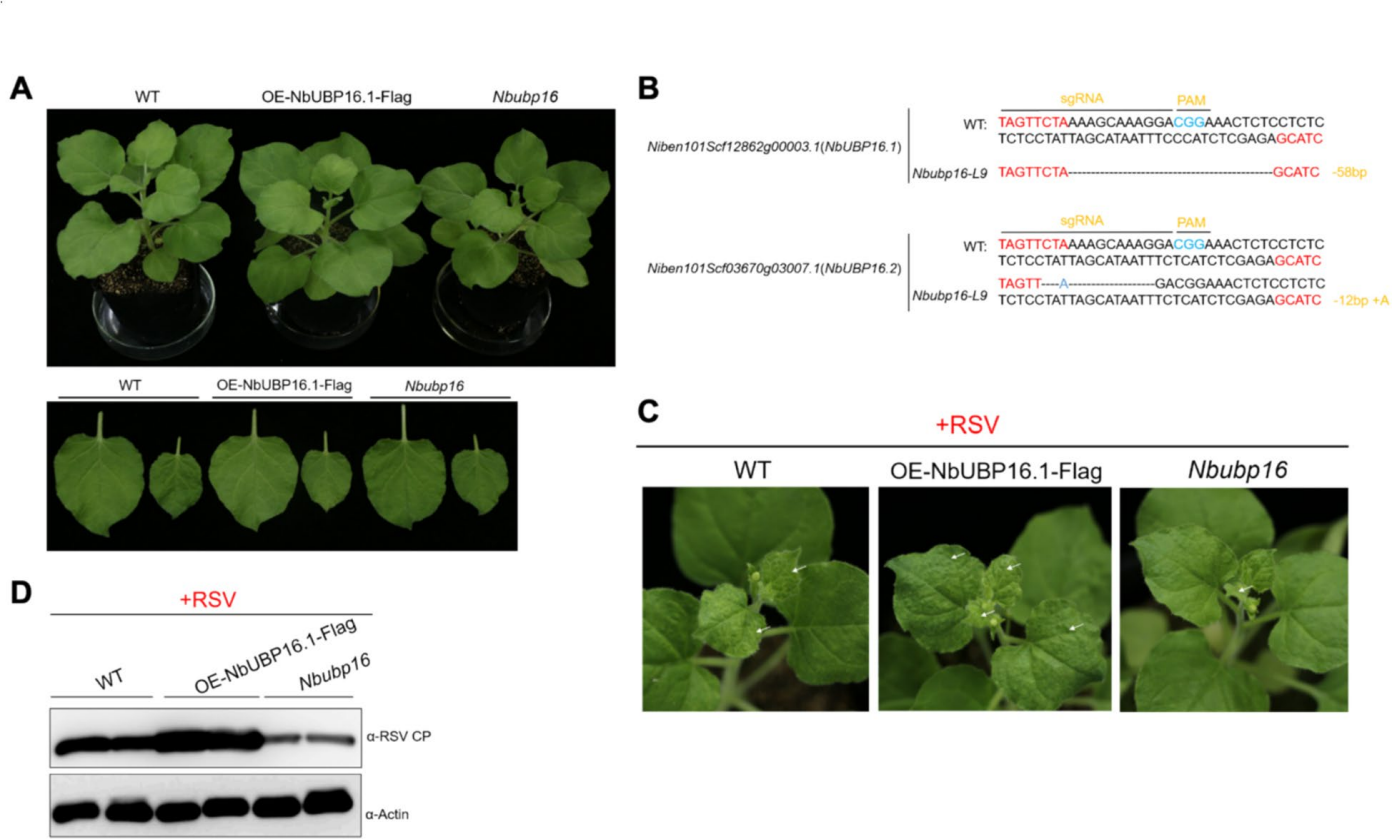
NbUBP16.1 positively regulates RSV infection. **A** Phenotypes of *NbUBP16.1* overexpressing (OE-NbUBP16.1), *Nbubp16* knockout *(Nbubp16)* and wild type *N. benthamiana* plants. **B** Schematic diagram of editing types of *Nbubp16* knockout *N. benthamiana* plants. **C** Photographs of representative RSV symptoms in WT, OE-NbUBP16.1 and *Nbubp16* N. benthamiana plants after RSV infection at 15 dpi. White arrows denote leaves with obvious symptoms. **D** RSV CP accumulation in wild-type, OE-NbUBP16.1 and *Nbubp16 N. benthamiana* plants infected by RSV at 15 dpi. Actin was used as loading controls

To further explore whether NbUBP16.1 regulates RSV infection by modulation the stability of NbSHMT1, we constructed overexpression vectors for NbUBP16.1, incorporating them into the pCambia vector to generate 2 × 35S-NbUBP16.1-Flag vectors. This vector was then transformed into *N. benthamiana* plants. The successful integration of the transgenes was confirmed by RT-PCR (Fig. S5). To create *NbUBP16* knockout plants, both *NbUBP16.1* and its allele *NbUBP16.2* were simultaneously knocked out by CRISPR/Cas9-mediated genome editing. Sanger sequencing confirmed the shift in the open reading frame (ORF) of *NbUBP16.1* and *NbUBP16.2* due to CRISPR/Cas9-mediate editing (Fig. 5B). The plant height and leaf size of OE-NbUBP16.1 and *Nbubp16* transgenic plants were morphologically indistinguishable from wild-type *N. benthamiana* plants under normal conditions (Fig. 5A). This is consistent with the phenotype of *AtUBP16* transgenic plant (Zhou et al. 2012).

To determine whether NbUBP16.1 can regulate RSV infection, OE-NbUBP16.1, *Nbubp16*, and wild-type *N. benthamiana* plants were challenged with RSV. The results showed that, compared with wild-type plants, OE-NbUBP16.1 transgenic plants exhibited enhanced viral symptoms (Fig. 4A, 5C and Fig. S6) and increased accumulation of the RSV capsid protein (Fig. 4E and 5D). In contrast, *Nbubp16* plants showed reduced viral symptoms and diminished RSV capsid protein accumulation (Fig. 5C, D and Fig. S6). These results demonstrated that NbUBP16.1 positively regulates RSV infection.

### NbUBP16.1 negatively regulates accumulation of ROS by modulating the stability of NbSHMT1

**Fig. 6:**
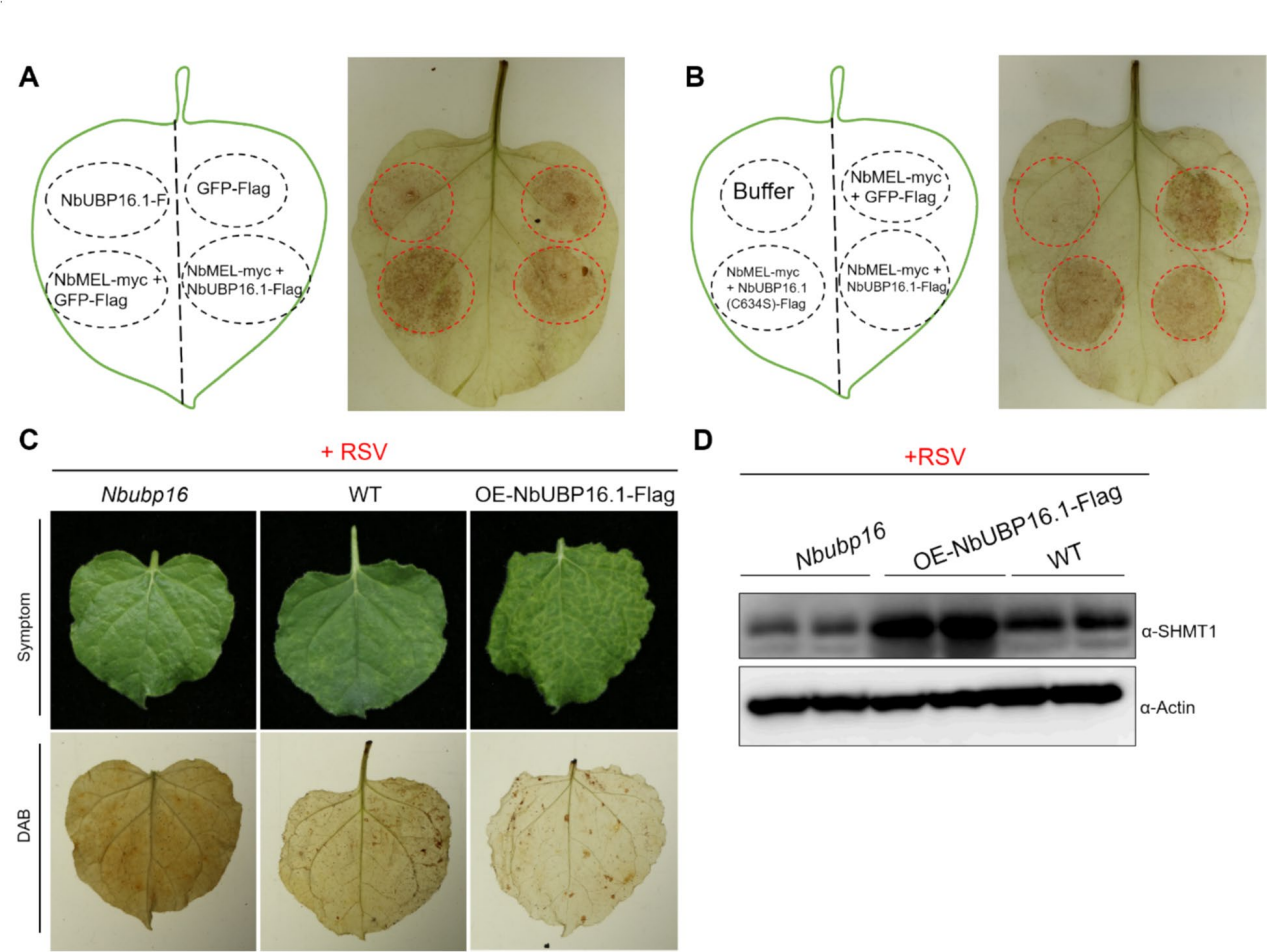
NbUBP16.1 negatively regulates accumulation of ROS by modulating the stability of NbSHMT1. **A** DAB staining of *N. benthamiana* leaves transiently expressing GFP-Flag, NbUBP16.1-Flag, NbMEL-Myc with GFP-Flag or NbUBP16.1-Flag by agroinfiltration. **B** DAB staining of *N. benthamiana* leaves transiently expressing buffer, NbMEL-Myc with GFP-Flag, NbMEL-Myc with NbUBP16.1-Flag or NbUBP16.1(C634S)-Flag by agroinfiltration. **C** DAB staining of wild-type, OE-NbUBP16.1 and *Nbubp16 N. benthamiana* leaves after RSV infection at 15 dpi. **D** NbSHMT1 accumulation wild-type, OE-NbUBP16.1 and *Nbubp16 N. benthamiana* plants after RSV infection at 15 dpi. Actin was used as loading controls

To further know whether NbUBP16.1 could regulate MEL-SHMT1 module-mediated ROS accumulation, we performed DAB staining assay by transiently expressing GFP-Flag, NbMEL-Myc, NbUBP16.1-Flag, or combinations of NbMEL-Myc with GFP-Flag, NbUBP16.1-Flag, or the enzyme activity-deficient mutant NbUBP16.1(C634S)-Flag in *N. benthamiana* leaves. The results showed that, compared with transient expression of NbMEL, which significantly induce ROS accumulation, the co-expression of NbUBP16.1 notably reduce ROS level (Fig. 6A). In contrast, the NbUBP16.1(C634S) mutant could not effectively suppress ROS accumulation (Fig. 6B). These results showed that NbUBP16.1 could regulate MEL-SHMT1 module-mediated ROS accumulation.

To detect whether NbUBP16.1 regulates the stability of NbSHMT1 protein and the accumulation of ROS after RSV inoculation, OE-NbUBP16.1, *Nbubp16*, as well as wild-type *N. benthamiana* plants were challenged with RSV. DAB staining and protein detection were performed at 15 dpi. The results showed that, compared with wild-type plants, OE-NbUBP16.1 transgenic plant exhibited increased NbSHMT1 protein accumulation and reduced ROS accumulation following RSV inoculation, while *Nbubp16* plants showed decreased NbSHMT1 protein levels and elevated ROS accumulation (Fig. 6C and D). These results further support the conclusion that NbUBP16.1 inhibits ROS accumulation by stabilizing NbSHMT1.

## Discussion

During the co-evolution of plants and plant viruses, plants utilize defense mechanisms such as RNAi and ETI to resist virus infection (Fang and Qi 2016; Chen et al. 2023; Wu et al. 2023), while plant viruses utilize various effector proteins to continuously interfere with plant defense responses and promote virus infection (Fu et al. 2018; Li et al. 2021; Ray and Casteel 2022).

Ubiquitination is an important pathway to regulate protein stability in eukaryotes, which plays an important role in plant resistance to pathogen infection (Copeland and Li 2019; Trujillo 2021; Langin et al. 2023). Ubiquitination dynamically regulates protein stability by a series of enzymes that ubiquitinate or deubiquitinate substrate proteins. Previously, many studies have reported that deubiquitinases in plants can participate in regulating plant physiological processes and abiotic stress. UBP12 and UBP13 play important roles in regulating epigenetic modifications and plant circadian clock (Derkacheva et al. 2016; Lee et al. 2019; Xiong et al. 2022). UBP3 and UBP4 are crucial in pollen development (Doelling et al. 2007). UBP15 regulates initiation of axillary meristems by forming a regulatory module with COTYLEDON2 (CUC2)/CU3-DA1 (Li et al. 2020). However, whether plant deubiquitinases can regulate plant virus infection has not been explicitly reported yet.

**Fig. 7:**
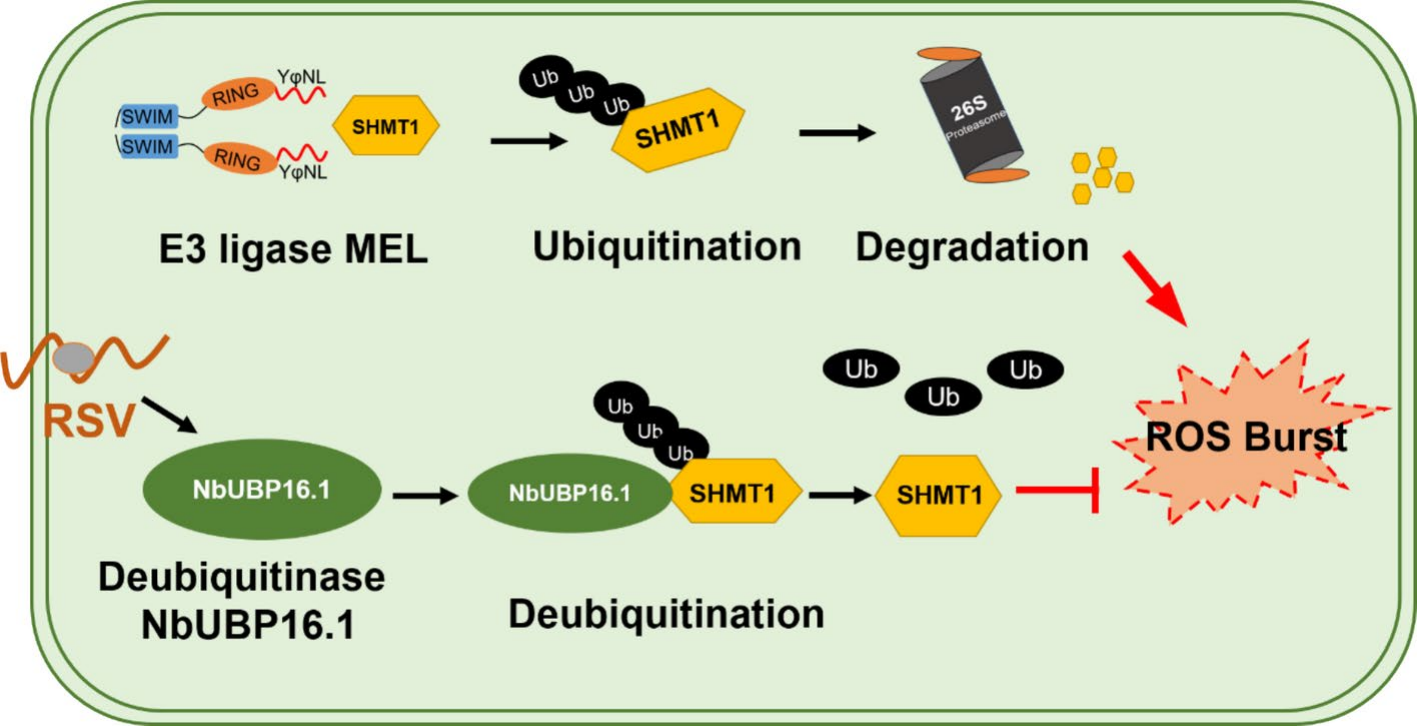
Working model for NbUBP16 promote RSV infection by stabilizing NbSHMT1 and inhibiting ROS accumulation. RSV infection can interfere with the plant deubiquitination pathway and specifically upregulate the expression of the deubiquitinase *NbUBP16*. NbUBP16.1 interacts with NbSHMT1 and removes polyubiquitination modification of NbSHMT1 mediated by E3 ligase NbMEL, which stabilizes NbSHMT1 and inhibits ROS burst, thereby promoting RSV infection

Previously, our group identified an evolutionarily conserved E3 ligase MEL, which activates plant defense responses by degrading SHMT1, core factor in the photorespiration pathway, thereby suppressing RSV infection. In this study, we found that the ubiquitination level of NbSHMT1 mediated by E3 ligase NbMEL increased significantly in the early stage of RSV infection. Moreover, we observed that RSV infection interferes with the plant deubiquitination pathway and specifically upregulate the expression of *NbUBP16*. Further study revealed that NbUBP16.1 can interact with NbSHMT1 and remove polyubiquitination modification of NbSHMT1 mediated by E3 ligase NbMEL and stabilize NbSHMT1, thereby inhibiting ROS burst and promoting RSV infection (Fig. 7). As far as we know, this is the first study about plant deubiquitinases involved in regulating plant virus infection. These findings highlight a novel viral strategy that manipulates host deubiquitination pathway to facilitate infection.

AtUBP16 has been reported to be involved in salt tolerance through positively regulating plasma membrane Na +/H + antiport activity and modulating AtSHMT1 stability and with *AtUBP16* knockout mutants exhibiting increased salt sensitivity. Additionally, Zhao et al. reported that UBP16 interacts with HEAVY METAL ASSOCIATED ISOPRENYLATED PLANT PROTIN27 (HIPP27) and plays an important role in cadmium detoxification (Zhao et al. 2013). Moreover, the ubp16 mutant showed more sensitive to cadmium than wild-type. However, our research found that UBP16 positively regulates RSV infection, as *Nbubp16* mutant plants showed more resistance to RSV infection. These results suggest that ubp16 may play different roles in plant biotic and abiotic stress processes, underscoring the dynamic balance of plant resistance to pathogen invasion and adaptation to environmental stress.

Interestingly, in our analysis of the transcriptome data of RSV infection, only *ubp16* was upregulated, while *ubp15* was significantly downregulated, and *ubp3*, *ubp4*, *ubp12*, and *ubp13* showed no significant changes. However, studies have reported that meristematic tissue plays an important role in the antiviral process, so we speculate that *ubp15* may also be involved in regulating RSV infection.

Due to the high homology of the 5 'and 3' ends of the coding regions of *NbUBP16.1* and *NbUBP16.2*, we only amplified the full-length *NbUBP16.1* from tobacco. Although the high homology (92%) of the amino acid sequences of NbUBP16.1 and NbUBP16.2, silencing either *NbUBP16.1* or *NbUBP16.2* can inhibit RSV infection, so we speculate that NbUBP16.2 may also play a role in regulating RSV infection. In the future, the function of *UBP16.2* and its relationship with *UBP16.1* can be explored.

## Conclusion

We analyzed RNA-sequencing data of RSV-infected *Nicotiana benthamiana* plants and found that RSV infection could modulate plant deubiquitination pathway and specifically upregulate the expression of the deubiquitinase *NbUBP16*. NbUBP16 stabilizes NbSHMT1 by binding to NbSHMT1 and removing its polyubiquitination modification mediated by E3 ligase MEL, which inhibits downstream SHMT1-mediated ROS accumulation and thereby facilitates RSV infection. Our findings provide new insights into the molecular arms race between pathogens and plants, demonstrating how a plant virus can undermine plant defenses by hijacking host deubiquitination pathways. Whether other plant deubiquitinases play a role in regulating the infection of different plant viruses deserves further exploration.

## Materials and methods

### Plant materials, plant transformation and agroinfiltration

The full-length ORFs of *NbUBP16.1*, *NbMEL* and *NbSHMT1* were amplified from RSV infected *N. benthamiana* cDNA generated by reverse transcription of total RNA. cDNA was synthesized using ReverTra Ace qPCR RT Master Mix with gDNA Remover (TOYOBO, Osaka, Japan) according to the manufacturer’s instructions. A point mutation of position 634, amino acids cysteine changed to serine, was introduced into *NbUBP16.1* by site-direct mutagenesis. All primers used for cloning in this study are listed in Supplemental Table 1.

*N. benthamiana* plants were grown in the chamber at 26 °C and 65% relative humidity under 16/8 h day/night conditions. For transgenic overexpression of *NbSHMT1* and *NbUBP16.1*, *Agrobacterium tumefaciens* EHA105 strains carrying pCambia-2*35s-NbSHMT1-Flag and pCambia-2*35s-NbUBP16.1-Flag were used for transformation using standard protocols as described by (Fu et al. 2022). RT-qPCR and western blot were performed to verify the gene expression.

### Histochemical staining of H_2_O_2_

Leaves were carefully transferred to 50 mL polypropylene tubes and completely covered with freshly prepared DAB solution (1 mg/mL 3, 3-diaminobenzidine, PH 3.8, Sigma) and then kept in the dark for 8–12 h. The DAB solution was discarded, then 95% ethanol was added and the tubes were incubated in the water bath at 100 °C to clear the chlorophyll (Bach-Pages and Preston 2018).

### Yeast two-hybrid assay

Y2H assay was performed as described previously (Fu et al. 2022).

### Co-IP assay

Co-IP assay was performed as described previously (Fu et al. 2022).

### In vivo ubiquitination assay

To detect whether NbUBP16.1 and NbUBP16.1(C634S) could interfere with ubiquitination of NbSHMT1 mediated by NbMEL In vivo, we transient co-expressed NbSHMT1-Flag, NbMEL-GFP, Ubiquitin-Myc, NbUBP16.1-Myc or NbUBP16.1(C634S)-Myc in *N. benthamiana* leaves, 100 uM CHX and 100 uM MG132 was pre-infiltrated into infiltrated leaves 12 h before total protein extraction. Total proteins were extracted and NbSHMT1-Flag was immunoprecipitated using anti-Flag beads. The poly-ubiquitination of NbSHMT1-Flag was detected with anti-Flag or anti-Ub antibody.

### BiFC assays

BiFC was performed as described previously (Fu et al. 2018). *A. tumefaciens* EHA105 strains carrying constructs to express 2YC/N-NbUBP16.1 or 2YC/N-GUS and 2YC/N-NbSHMT1 were co-infiltrated into *N. benthamiana* leaves. After expressed for 36–48 h, *N. benthamiana* epidermis tissues were observed under the confocal microscope (Zeiss 880).

### Immunoblotting and antibodies

Proteins were probed with primary anti-Flag (Sigma-Aldrich, Cat#:M8823); anti-MYC (Vazyme, Cat#:Ab307-02); anti-Actin (ABclonal, Cat#:AC009); anti-SHMT (Agrisera, Cat#:AS05 075); anti-Ub (Agrisera, Cat#:AS08 307A), followed by the corresponding secondary antibodies conjugated to horseradish peroxidase [Goat anti-Rabbit IgG (H + L) Secondary Antibody, HRP (Thermo, Cat#:31460); Goat anti-Mouse IgG (H + L) Secondary Antibody, HRP (Thermo, Cat#:31430)]. Blotted signal was visualized using chemiluminescence according to the manufacturer’s manual (GE Healthcare, USA).

## Supplementary Information

Additional file 1: Fig. S1. Detection of transcripts of *NbSHMT1* in OE-NbSHMT1-Flag or wild type *N. benthamiana* leaves by qRT-PCR. Fig. S2. NbSHMT1-Flag accumulation in wild-type and *NbSHMT1* overexpression transgenic *N. benthamiana* plants. Fig. S3. DAB staining of wild-type and OE-NbSHMT1 *N. benthamiana* leaves. Fig. S4. Detection of transcripts of *NbMEL* and *NbUBP16.1* in wild type *N. benthamiana* leaves after RSV infection by qRT-PCR. Fig. S5. Detection of transcripts of *NbUBP16.1* in OE-NbUBP16.1-Flag transgenic *N. benthamiana* leaves by qRT-PCR. Fig. S6. Photographs of representative RSV symptoms in WT, OE-NbUBP16.1 and *Nbubp16 N. benthamiana* plants after RSV infection at 15 dpi. Table S1. Primers used in the study.

## Data Availability

The data that support the findings of this study are available from the corresponding author upon reasonable request.

## Abbreviations

WT: Wild-type
RSV: Rice stripe virus
SHMT1: Serine hydroxymethyltransferase 1
ROS: Reactive oxygen species
MAPK: Mitogen-activated protein kinase
PTI: PAMP-triggered immunity
ETI: Effector-triggered immunity
PR1: Pathogenesis-related protein 1
UBP: Ubiquitin-specific protease
TRV: Tobacco rattle virus
CP: Coat protein
