# Supplementary material for: Upregulation of deubiquitinase UBP16 induced by rice stripe virus infection stabilizes SHMT1 to suppress ROS accumulation and facilitate virus infection in Nicotiana benthamiana
Source: Stress Biol. 2025 Oct 26;5(1):62. doi: 10.1007/s44154-025-00265-2 (PMC12553715; doi:10.1007/s44154-025-00265-2)
Supplement: Supplementary file 1 — Additional file 1: Fig. S1. Detection of transcripts of NbSHMT1 in OE-NbSHMT1-Flag or wild type N. benthamiana leaves by qRT-PCR. Fig. S2. NbSHMT1-Flag accumulation in wild-type and NbSHMT1 overexpression transgenic N. benthamiana plants. Fig. S3. DAB staining of wild-type and OE-NbSHMT1 N. benthamiana leaves. Fig. S4. Detection of transcripts of NbMEL and NbUBP16.1 in wild type N. benthamiana leaves after RSV infection by qRT-PCR. Fig. S5. Detection of transcripts of NbUBP16.1 in OE-NbUBP16.1-Flag transgenic N. benthamiana leaves by qRT-PCR. Fig. S6. Photographs of representative RSV symptoms in WT, OE-NbUBP16.1 and Nbubp16 N. benthamiana plants after RSV infection at 15 dpi. Table S1. Primers used in the study. [file 44154_2025_265_MOESM1_ESM.pdf]

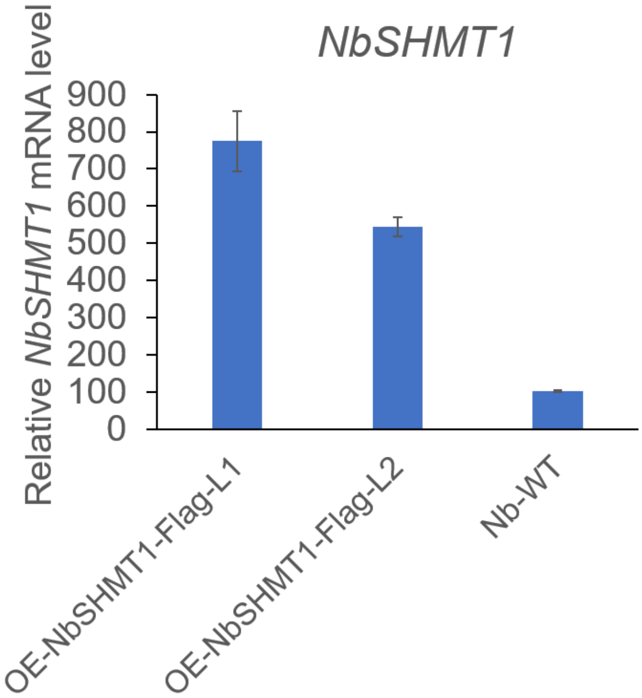

Fig. S1 Detection of transcripts of *NbSHMT1* in OE-NbSHMT1-Flag or wild type *N. benthamiana* leaves by qRT-PCR. Actin was used as reference gene.

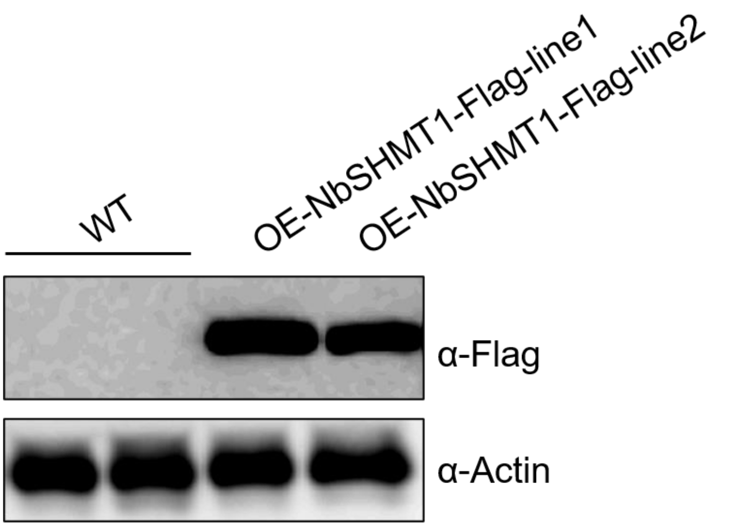

Fig. S2 NbSHMT1-Flag accumulation in wild-type and *NbSHMT1* overexpression transgenic *N. benthamiana* plants.

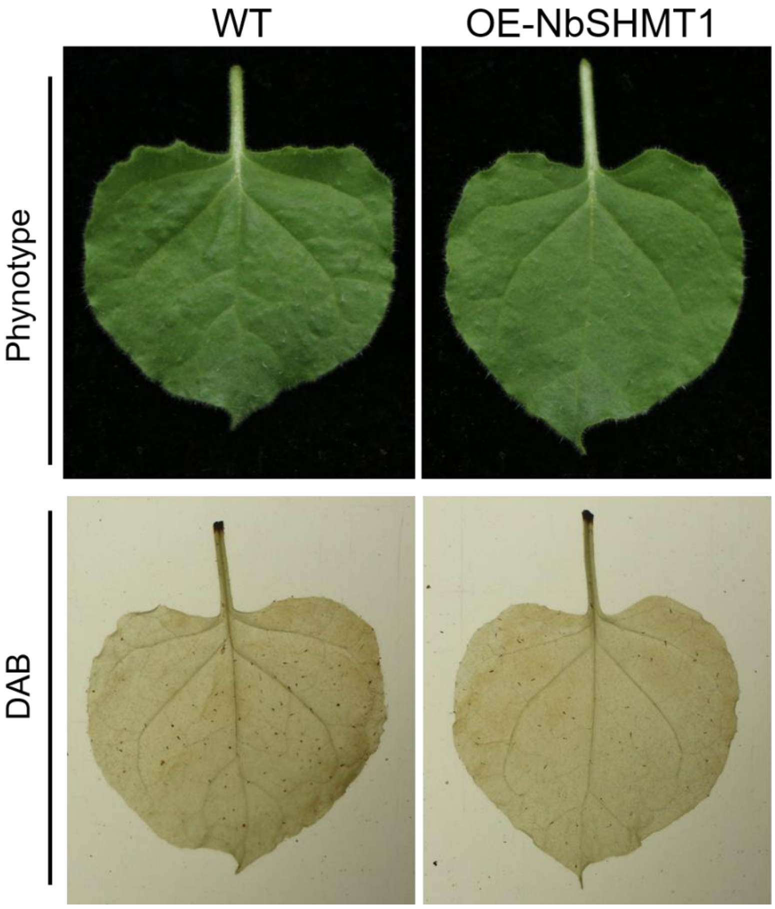

Fig. S3 DAB staining of wild-type and OE-NbSHMT1 *N. benthamiana* leaves.

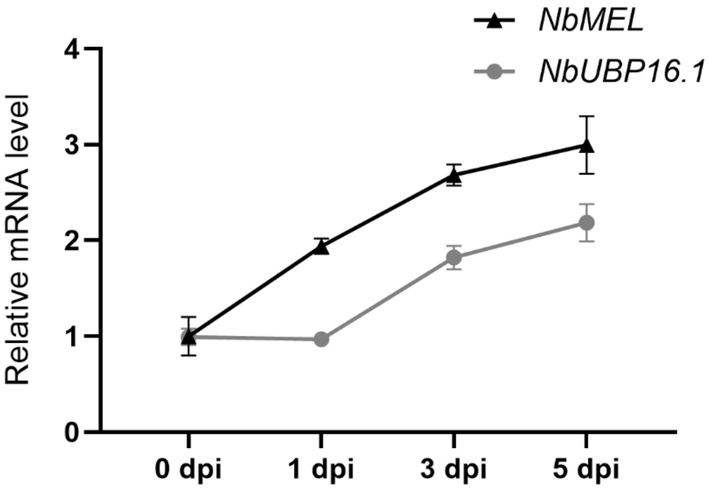

Fig. S4 Detection of transcripts of *NbMEL* and *NbUBP16.1* in wild type *N. benthamiana* leaves after RSV infection by qRT-PCR. dpi, days post inoculation. Actin was used as reference gene.

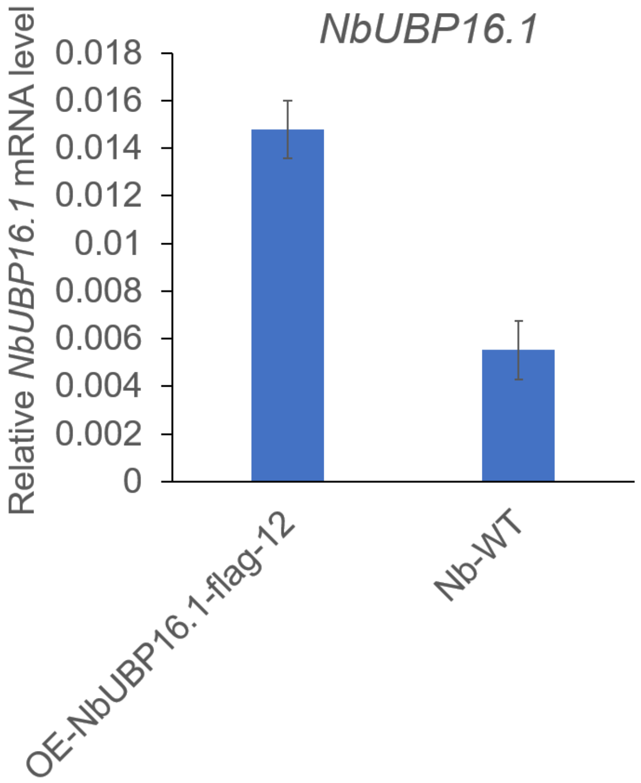

Fig. S5 Detection of transcripts of *NbUBP16.1* in OE-NbUBP16.1-Flag or wild type *N. benthamiana* leaves by qRT-PCR. Actin was used as reference gene.

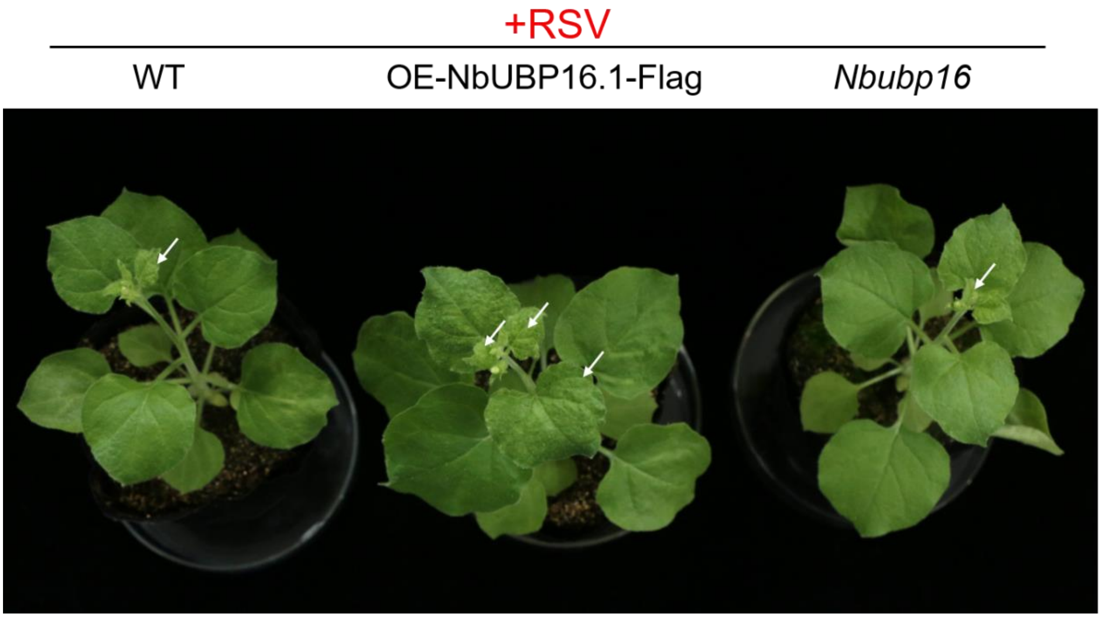

Fig. S6. Symptoms in WT, OE-NbUBP16.1 and *Nbubp16 N. benthamiana* plants after RSV infection at 15 dpi. White arrows represent leaves with obvious symptoms.

Table S1 Primers used in this study

Primer Name	Primer sequence (5'-3')	Purpose
2*35S-NbUBP16.1-Flag-F	acgggggacgagctcggtaccATGCTCGTAGGAGGGGATCTAGG	Construct 2*35S-NbUBP16.1-Flag
2*35S-NbUBP16.1-Flag-R	gtggtccttatagtcgtcgacTCGATCTCTACAAGTTGCAGGAGA	Construct 2*35S-NbUBP16.1-Flag
2*35S-NbUBP16.1-Myc-F	acgggggacgagctcggtaccATGCTCGTAGGAGGGGATCTAGG	Construct 2*35S-NbUBP16.1-Myc
2*35S-NbUBP16.1-Myc-R	gagtttctgctccatgtcgacTCGATCTCTACAAGTTGCAGGAGA	Construct 2*35S-NbUBP16.1-Myc
2*35S-NbSHMT1-Flag-F	acgggggacgagctcggtaccATGGCCATGGCAACGGCT	Construct 2*35S-NbSHMT1-Flag
2*35S-NbSHMT1-Flag-R	gtggtccttatagtcgtcgacTTTTTTGTACTTCATGGTTTCCTTCT	Construct 2*35S-NbSHMT1-Flag
BD-NbUBP16.1-F	atggccatggaggccgaattcATGCTCGTAGGAGGGGATCTAGG	Construct BD-NbUBP16.1
BD-NbUBP16.1-R	cgacggatccccgggaattcTCATCGATCTCTACAAGTTGCAGG	Construct BD-NbUBP16.1
2YN/2YC-NbUBP16.1-F	atttacgaacgatagttaattaaATGCTCGTAGGAGGGGATCTAGG	Construct BIFC-NbUBP16.1
2YN/2YC-NbUBP16.1-R	acctcctccactagtggcgcgccCTCGATCTCTACAAGTTGCAGGAG	Construct BIFC-NbUBP16.1
pTRV2-NbUBP16.1-F	agaaggcctccatggggatccGGAAACCAGTCGATCGAGCTG	Construct pTRV2-NbUBP16.1
pTRV2-NbUBP16.1-R	cgtgagctcggtaccggatccAAGATTCTTTTTCTCTTTCTTTTGGC	Construct pTRV2-NbUBP16.1
pTRV2-NbUBP16.2-F	agaaggcctccatggggatccAGAAACCAGTTGATCGAGCTGAA	Construct pTRV2-NbUBP16.2
pTRV2-NbUBP16.2-R	cgtgagctcggtaccggatccGAGAAGAATCTTTTTGTCTATCTTTTGG	Construct pTRV2-NbUBP16.2
Oligo A-CRISPR-NbUBP16-sgRNA	TGATTGTAGTTCTAAAAGCAAAGGA	Construct CRISPR-NbUBP16-sgRNA
Oligo B-CRISPR-NbUBP16-sgRNA	AAACTCCTTTGCTTTTAGAACTACA	Construct CRISPR-NbUBP16-sgRNA
Detection-sgRNA-NbUBP16.1-F	GATAACATACCTATTATGGCGAACA	Detection editing efficiency of NbUBP16.1
Detection-sgRNA-NbUBP16.1-R	CTCACAAAATGTGTATATATTTCCTC	Detection editing efficiency of NbUBP16.1
Detection-sgRNA-NbUBP16.2-F	GTGTCGATAACATACCTATTAAGTT	Detection editing efficiency of NbUBP16.2
Detection-sgRNA-NbUBP16.2-R	CTTGCATTTTTAGCATTCTTGCA	Detection editing efficiency of NbUBP16.2
PM-NbUBP16.1(C634S)-F	GTAACAGCtccTATGCTAATGCTGTGCTTCAATGC	Construct NbUBP16.1 point mutant
PM-NbUBP16.1(C634S)-R	AGCATAggaGCTGTTACCACAGTTTACGAGGC	Construct NbUBP16.1 point mutant
qPCR-NbUBP16.1-F	CGAGCTGAATCAAGATTCGAAGTTA	RT-qPCR
qPCR-NbUBP16.1-R	CTCTTTCTTTTGGCAATTGTGAAAC	RT-qPCR

qPCR-NbUBP16.2-F	AGAAACCAGTTGATCGAGCTGAA	RT-qPCR
qPCR-NbUBP16.2-R	GAGAAGAATCTTTTTGTCTATCTTTTGG	RT-qPCR
qPCR-NbACTIN-F	CAATCCAGACACTGTACTTTCTCTC	RT-qPCR
qPCR-NbACTIN-R	AAGCTGCAGGTATCCATGAGACTA	RT-qPCR
qPCR-NbPR1-F	AGGTGTGTGGACACTATACTCAAGTG	RT-qPCR
qPCR-NbPR1-R	CACATATAACGTGAAATGGACGC	RT-qPCR
qPCR-NbPR2-F	GGAGATTATTGTATCTGAAAGTGGATG	RT-qPCR
qPCR-NbPR2-R	TCCTTCCTTATTATTTTCATCAAATATG	RT-qPCR
qPCR-NbPR5-F	TACATTCACATGTTCTAATGCCAATT	RT-qPCR
qPCR-NbPR5-R	GACCCTGATTCAGTACCTGGAAGT	RT-qPCR
qPCR-NbMEL-F	CACATCCTCTTCGTCCTCATTC	RT-qPCR
qPCR-NbMEL-R	CTTAAAAAACGTCTCGTGAAATCT	RT-qPCR
